# A meta-analysis on the prevalence of resistance of *Staphylococcus aureus* to different antibiotics in Nigeria

**DOI:** 10.1186/s13756-023-01243-x

**Published:** 2023-04-25

**Authors:** Christian Kelechi Ezeh, Chibuzor Nwadibe Eze, Marie Esther Uju Dibua, Stephen Chijioke Emencheta

**Affiliations:** 1 Department of Microbiology, University of Nigeria, Nsukka, Enugu State Nigeria; 2 Department of Pharmaceutical Microbiology, University of Nigeria, Nsukka, Enugu State Nigeria

**Keywords:** Antibiotic resistance, Meta-analysis, Nigeria, *Staphylococcus aureus*

## Abstract

**Background:**

Rapid emergence of multidrug resistant *Staphylococcus aureus* has resulted to difficulty in treatment of infections caused by such strains. The aim of this meta-analysis study was to determine the pooled prevalence of resistance of *S. aureus* to different antibiotics in Nigeria.

**Methods:**

Literature search for studies was done using Google scholar, PubMed, Science direct, and African Journal Online. The prevalence of *S. aureus* resistance to different antibiotics was evaluated using the meta-analysis proportion command in MedCalc software version 20.0 adopting a rand effect model. I^2^ statistic and Egger test in MedCalc was used to evaluate the heterogeneity and the presence of publication bias among studies respectively.

**Results:**

A total of 40, 682 studies were retrieved through the database search of which 98 studies met the study inclusion criteria. Prevalence of resistance of *S. aureus* to different antibiotics ranges from 13 to 82%. Results showed a very high degree of resistance to penicillin G (82% [95% confidence interval (CI) 61%, 0.96%]), cloxacillin (77% [95% CI 64%, 88%]), amoxacillin (74% [95% CI 66%, 81%]), cefuroxime (69% [95% CI 51%, 85%]), ampicillin (68% [95% CI 53%, 81%]). Moderately resistance to erythromycin (47% [95% CI 40%, 53%]), chloramphenicol (47% [95% CI 37%, 56%]), methicillin (46% [95% CI 37%, 56%]), ofloxacin (24% [95% CI 18%, 31%]) and rifampicin 24% [95% CI 6%, 48%]). Low resistance was observed in vancomycin 13% (95% CI 7%, 21%). For each individual meta-analysis, high heterogeneity was observed with I^2^ range (79.36–98.60%) at p-values ≤ 0.01). Egger’s tests for regression intercept in funnel plots indicated no evidence of publication bias.

**Conclusion:**

This meta-analysis study established that *S. aureus* in Nigeria has developed resistance to commonly used antibiotics such as the beta-lactam class antibiotics, sulphonamides, tetracyclines, chloramphenicol, and vancomycin. Hence it is imperative to develop programs to promote rational use of antimicrobial agents, infection prevention and control to reduce the incidence of antimicrobial resistance.

**Supplementary Information:**

The online version contains supplementary material available at 10.1186/s13756-023-01243-x.

## Background

*Staphylococcus aureus* (*S. aureus*) is well adapted to various environments due to their metabolic versatility and pharmic resistance ability. *S. aureus* colonize the skin and nasopharyngeal membranes as normal microbiota in healthy individuals [1]. However, they cause myriad of detrimental infections when they invade the internal tissues or enter the bloodstream. *S. aureus* is an important pathogen involved in both hospital-acquired and community-acquired infections and causes many infectious diseases ranging from mild skin and soft tissue infections, bones and joint infections, infective endocarditis, cardiovascular disorders, osteomyelitis, bacteremia, and fatal pneumonia in both healthy and individuals with underlying diseases [2]. The high incidence of both community and nosocomial staphylococcal infections coincide with the emergence of multidrug resistant *S. aureus* which renders antibiotic treatments ineffective [3].

*S. aureus* has become resistant to various antibiotics over the past years especially to the beta-lactam class of antibiotics [4]. Emergence of methicillin resistant *S. aureus* (MRSA) and vancomycin resistant *S. aureus* (VRSA) constitutes a serious global public health problem. Currently, VRSA and MRSA strains are classified as very potent and dangerous agents that can potentially cause devastating damage worldwide in the absence of effective treatment options [5].

Various mechanisms of resistance utilized by *S. aureus* include: production of beta-lactamase enzymes to deactivate beta-lactam antibiotics, efflux pump for extruding antibiotics such as tetracyclines [6], reduced accumulation of macrolides antibiotics [7], production of aminoglycosides modifying enzymes to inactivate aminoglycoside antibiotics, alteration of DNA gyrase and topoisomerase IV expression of floroquinolones antibiotics, and expression of Mec genes which alters penicillin binding proteins [8].

In Nigeria, the prevalence of multi-drug resistant pathogens continue to be on the increase due to several factors such as drug misuse, self medication, lack of trained medical personnel, and poverty. As the world battles the persistent rise in antimicrobial resistance (AMR), it is pertinent that adequate data and information about AMR is known which can serve as the basic foundation for setting out effective interventions to contain the crisis of AMR. From the literature, no prior meta-analysis has been done on *S. aureus* resistance to different antibiotics routinely use in Nigeria. Due to the various infections caused by *S. aureus*, it is pertinent to determine the pooled prevalence of resistance of *S. aureus* to various routinely used antibiotics in Nigeria. This will help in improving treatment options and enlighten the populace on the menace and the possible cause of treatment failures due to the increasing rise of multidrug resistant strains. The aim of this meta-analysis was to determine the pooled prevalence of *S. aureus* resistance to various routinely used antibiotics in Nigeria.

## Methods

### Study design

Meta-analysis was adopted to evaluate the prevalence of *S. aureus* of resistance to various antibiotics in Nigeria using the appropriate studies that rely solely on *S. aureus* from the title. The prevalence of resistance of *S. aureus* to various routinely used antibiotics in Nigeria is a country wide study as it covers studies from the six geo-political regions of Nigeria. Meta-analysis was adopted because it is a quantitative study of pooled prevalence of resistance of *S. aureus* to routinely use antibiotics in Nigeria.

### Search strategy

Electronic search engines including Google scholar, PubMed, ScienceDirect, and African Journal Online (AJOL) were used to search for available studies from 23rd March to May 2022. Relevant key words such as Staphylococcus, antibiotic resistance, antibacterial resistance, antimicrobial resistance, drug resistance, drug susceptibility, Nigeria were used during the search. These key words were used in different combinations (Staphylococcus OR *S. aureus* AND antibiotic resistance OR antibacterial resistance OR antimicrobial resistance OR drug resistance AND Nigeria) in various electronic databases using the Boolean operators. The reference lists of included articles were also check to identify studies relevant to the current study.

### Inclusion and exclusion criteria

The titles of search results of all retrieved articles were screened independently by two authors with the aim of including studies that address the research question. The articles were inserted into Zotero version 5.0.95.1 referencing application which helped in detecting duplicate articles. The title of the study which solely focused on prevalence of antimicrobial resistance of *S. aureus* was grouped as eligible for inclusion. *S. aureus* resistance in any state in Nigeria and studies only done in Nigeria represented in the title is the first criteria for inclusion. However, studies that focused on many microbial strains antimicrobial resistance were excluded.

In general, retrieved studies selected from predefined criteria were screened further using the inclusion criteria: studies that were research articles and used cross sectional design, studies that used human samples, studies that conducted antimicrobial susceptibility tests using the Clinical Laboratory Standard Institute (CLSI) guidelines, studies written in English language and studies with full text.

Exclusion criteria in this meta-analysis include: studies conducted on non-human samples, studies with isolates below 20, duplicate studies, studies that did not conduct antimicrobial susceptibility tests using the Clinical Laboratory Standard Institute (CLSI) guidelines studies not written in English, and review articles.

### Data extraction

Relevant data such as name of author (s) and publication year, study design, study place, clinical sample size, isolate source, total number of *Staphylococcus aureus* isolates tested in each research article, and total No. of isolates resistant each antibiotics. In situations where the proportion of susceptible isolates was reported, then the No. of resistant *Staphylococcus aureus* isolates was calculated by subtracting the percentage susceptibility from 100 and then dividing the result by 100 and multiplying to the total number of isolates. However, in situation where the proportion of the resistant isolates was given, then the No. of resistant *Staphylococcus aureus* isolates was calculated by dividing the proportion of the resistant isolates by 100 and multiply with the total number of isolates. The formula is given as thus:

1 $${\text{Prevalence}}\;{\text{of}}\;{\text{resistance}}\left( \% \right) = \frac{{number\;of\;resistant\;isolates}}{{total\;number\;of\;isolates}} \times 100$$

To ascertain the reporting of all relevant information in this meta-analysis, we followed the Preferred reporting Items for Systematic Review and Meta-analysis (PRISMA) [9] (Additional file 1: S1) guidelines.

### Statistical analysis procedures

In this meta-analysis, statistical analyses were performed using MedCalc statistical software version 20.0.1. The pooled prevalence of antibiotic resistance of *S. aureus* was evaluated using the meta-analysis proportion command in MedCalc. A total of 23 separate meta-analyses were carried out to evaluate the pooled prevalence of *S. aureus* resistance to 23 different antibiotics. Between 6 and 77 studies were included in the 23 different meta-analyses. I^2^ statistic command in MedCalc was used to evaluate the heterogeneity among the included studies. Random effect and fixed effect are two models used to estimate pooled prevalence in meta-analysis. In this study, due to the characteristically high heterogeneity of the included studies, the random effect model was used for meta-analysis at 95% CIs. Egger test was employed for assessing the presence of publication bias [10].

The Freeman-Tukey double arcsine transformation was used to ensure studies which report proportions near or at 0 and 1 were not being excluded. In addition, studies that report unusually high prevalence of resistance when compared to others, a sensitivity analysis was perform by removing the studies. If the point estimate of pooled prevalence after removing a study that reported unusually high prevalence of resistance lies within the 95% CI of the overall pooled estimate for all studies combined, the study is considered as having no significant influence on the overall estimate and vice versa.

## Results

### Characteristics of included studies

**Fig. 1:**
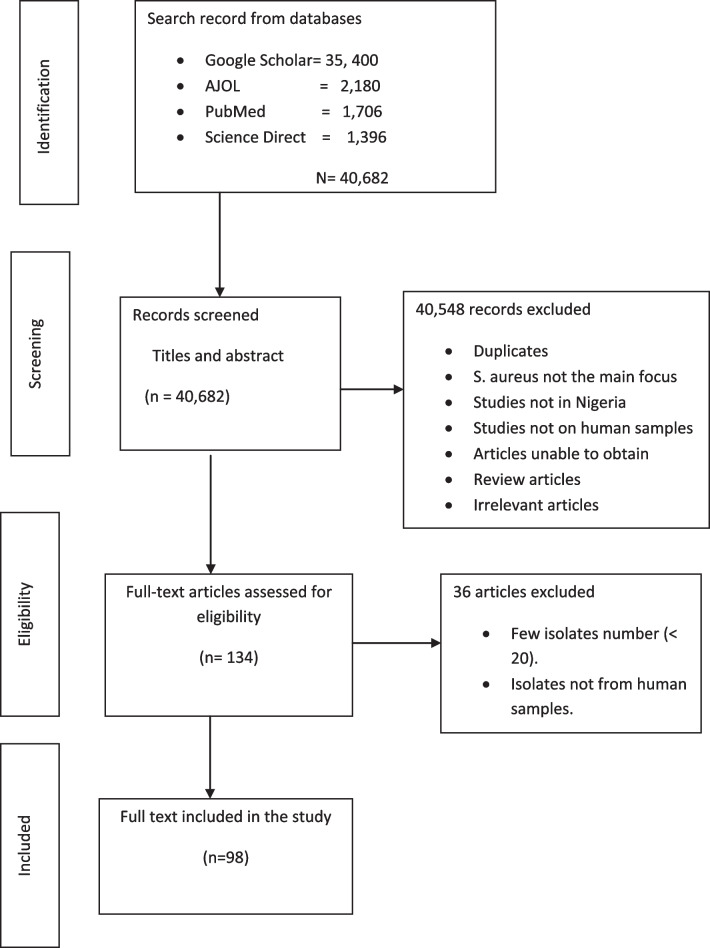
PRISMA flowchart for the selection and screening of eligible studies

Studies search record from electronic databases yielded 40, 682 of which 35, 400, 2, 180, 1,706, and 1396 were from Google scholar, AJOL, PubMed, and Science Direct, respectively. Articles from Google Scholar gave 35,400 results comprising of many studies irrelevant or that does not fit to the study aim; hence, they were screened randomly from titles alone. Screening of the titles reduced the number of eligible articles to 134 for full text assessment. After going through the full texts, 36 articles were excluded (reported small number of isolates and isolates not from human samples). Thus, 98 studies met the inclusion criteria of the study (Fig. 1).

About 46, 640 *S. aureus* isolates were tested against different antibiotics and 23,048 isolates were resistant to various antibiotics. Isolates sources include: nasal, blood, vaginal, ear, wound, urine, throat, pimples, hand, and mixed samples were collected from both symptomatic patients [61] and asymptomatic people [37]. Eighty six studies used primary data while twelve used records from hospitals. The characteristics of each study included is summarized Table 1.

**Table 1:** Characteristics of included studies

Reference	Study	Study place	Data type	Setting and sample source	Sample size	No of recovered isolates	Antibiotics used
[11]	Akortha and Ikenebomeli, 2010	South south (Benin)	Primary,	Hospital: Nasal	52	20	CPR, TET, CHL, ERY, AMP, OFL
[12]	Idris et al., 2018	Northwest (Kano)	Primary	Hospital: Blood		195	MET, CPR, TET, ERY, GEN, CLIN, CEF
[13]	Stanley et al., 2013	Southsouth (Porthacourt)	Primary	Hospital: Vaginal swab	265	74	MET, CPR, TET, ERY, AMP, GEN
[14]	Odu and Okonkwo, 2012	Southsouth (Porthacourt)	Primary	Urban:Nasal	100	32	MET, CIPRO, TET, ERY, AMP, GEN, CLIN, CXC, COT, STR
[15]	Isibor and Otabor, 2014	Southsouth (Edo)	Primary	Urban: Nasal	100	32	AMO, CTR, CRX
[16]	Nworie, 2013	Southeast (Ebonyi)	Primary	Urban: Nasal	87	20	VAN, CPR, TET, ERY, AMP, OFL, GEN, COT, CTR
[17]	Egbuobi et al., 2014	Southeast (Imo)	Primary	Hospital: Different clinical samples	200	76	MET
[18]	Olowo-Okere et al., 2017	Northwest	Primary	Hospital: Wound	38	20	CPR, ERY, AMO, GEN, NOR
[19]	Olorode et al. 2021	Southsouth (Bayelsa)	Primary	Hospital: Different clinical samples	250	25	MET, CPR, CHLERY, AMP, AMO, GEN, RIF, STR, NOR
[20]	Onanuga and Awhowho, 2012	Southsouth (Bayelsa)	Primary	Hospital: Urine	200	46	VAN, CPR, TET, CHL, AMP, OFL, GEN, COT, AUG, CRX, CEF
[21]	Ayodeji and Omoniyi, 2009	Southwest (Ogun)	Primary	Hospital; Different clinical samples	107	107	VAN, CPR, TET, ERY, AMP, AMO, GEN, CXC, COT, STR, CAZ, PEN
[22]	Onanuga and Onaolapo, 2008	Nortwest (Kaduna)	Primary	Urban: Urine	150	54	VAN, MET, CPR, AMP, OFL, GEN, CLIN
[23]	Chigbu and Ezeronye, 2003	Southeast (Abia)	Primary	Hospital: Ear and nasal	70	38	CPR, TET, CHL, ERY, AMP, AMO, GEN, RIF, CXC, PEN
[24]	Enabule et al., 2007	Southsouth	Primary	Hospital: Urine		80	CPR, TET, ERY, AMP, GEN
[25]	Yah et al., 2009	Southsouth (Benin)	Primary	Hospital: Wound	153	86	CPR, TET, CHL, ERY, GENCXC, COT
[26]	Onwubiko and Saidiq, 2011	Northwest (Kano)	Secondary	Hospital: Different clinical samples		150	CPR, TET, ERY, AMP, AMO, OFL, GENCXC, STR, PEN, CAZ
[27]	Onanuga and Temedie, 2011	SOuthsouth	Primary	Urban: Nasal	120	40	VAN, CPR, CHL, ERY, AMP, AMO, OFL, AUG, CRX, CEF
[28]	Onanuga et al., 2005	Northcentral (Abuja)	Primary	Hospital: Urine	150	60	VAN, MET, CPR, AMP, OFL, GEN, CLIN
[29]	Akanbi and Mbe, 2013	Northcentral (Abuja)	Primary	Hospital: Different clinical samples		214	VAN, MET, ERY, AMP, OFL, GEN
[30]	Terry et al., 2011	Nortwest	Secondary	Hospital: Different clinical samples		194	MET, TET, CHL, ERY, AMP, GEN, STR, CAZ, PEN, CTR
[31]	Iroha et al., 2012	Southeast (Ebonyi)	Primary	Hospital: Nasal		105	VAN, CPR, ERY, CLIN, CXC, COT, PEN
[32]	Eke et al., 2012	Southsouth (Edo)	Primary	Urban: Nasal and ear	100	39	MET, CPR, TET, AMP, PEN
[33]	Ekundayo and Ndubuisi, 2015	Southeast (Abia)	Primary	Hospital: Different clinical samples	100	113	TET, CHL, ERY, AMP, GEN, CXC, COT, AUG, STR, PEN
[34]	Obasuyi and Akerele, 2015	Southsouth (Edo)	Secondary	Hospital: Different clinical samples		75	MET
[35]	Akerele et al., 2015	Southsouth (Edo)	Primary	Urban: Nasal	200	99	MET, CPR, ERY, AMP, AMO, GEN, STR, CTR
[36]	Badger-Emeka et al., 2014	Southeast 9Enugu)	Primary	Hospital: Wound	34	34	VAN, MET, TET, CHL, ERY, AMO, OFL, GEN, CXC, COT, AUG, STR
[37]	Ayeni et al., 2015	Southsouth (Bayelsa)	Secondary	Urban: Nasal	185	185	ERY, AMP, PEN, CTR, NOR
[38]	Torimino et al., 2012	Southwest (Oyo)	Primary	Urban: Different clinical samples	50	40	CPR, TET, CHL, ERY, AMO, OFL, GENCXC, COT, STR, CTR
[39]	Bale et al., 2019	Southwest (Kwara)	Primary	Urban: Nasal	113	42	TET, ERY, OFL, CXC, AUG, CTR, CTR
[40]	Adesoji et al., 2019	Nortwest (Katsina)	Primary	Urban: Different clinical samples	120	120	ERY, OFL, GEN, CXC, AUG, CAZ, CRX, CTR
[41]	Ariom et al., 2011	Southeast (Ebonyi)	Primary	Hospital: Different clinical samples	709	84	MET, CPR, TET, GEN, CAZ, PN
[42]	Ajani et al., 2020	Southwest (Ogun)	Primary	Urban: Nasal	200	20	MET
[43]	Olonrunfemi et al., 2020	Northcentral	Primary	Urban: Urine	217	73	MET
[44]	Onanuga et al., 2021	Northeast	Primary	Urban: Nasal	262	46	TET, ERY, AMO, GENCOT
[45]	Ramalan et al., 2020	Northcentral (Nasarawa)	Primary	Hospital: Urine	202	62	CPR, CHL, ERY, AMP, AMO, GEN, STR
[46]	Udobi et al., 2013	Northwest (Kaduna)	Primary	Hospital: Skin and wound	217	69	CPR, AMO, GEN, CTR
[47]	Obasola et al., 2010	Southwest (Oyo)	Primary	Urban: Different clinical samples	50	50	TET, CHL, ERY, AMO, GENCXC, COT, AUG
[48]	Moses et al., 2017	Southsouth (Uyo)	Primary	Hospital: Nasal	130	41	VAN, CPR, TET, ERY, GENCLIN, CEF
[49]	Nsofor et al., 2015	Southeast (Imo)	Primary	Urban: Nasal	270	152	TET, CHL, ERY, GEN
[50]	Adetayo et al., 2014	Southwest (Oyo)	Primary	Hospital: Different clinical samples	150	66	VAN
[51]	Ejikeugwu et al., 2018	Southeast (Ebonyi)	Secondary	Hospital: Different clinical samples		39	ERY, GEN, CLIN, CXC, CEF
[52]	Anucha et al., 2021	Southeast (Anambra)	Primary	Hospital: Urine	236	62	VAN, TET, ERY, AMO, OFL, GEN, CRX
[53]	Agwu et al., 2010	Southsouth (Edo)	Primary	Hospital: Wound	220	66	VAN, RIF, CRX, CTR
[54]	Adesida et al., 2016	Southwest (Lagos)	Primary	Urban: Nasal	230	50	ERY, AMO, OFL, GEN, CXC, CAZ, CRX, CTR
[55]	Mofolorunsho et al., 2015	Northcentral (Kogi)	Primary	Hospital: Different clinical samples	100	22	CPR, TET, ERY, AMO, OFL, GEN, COT, STR
[56]	Osiyemi et al., 2018	Southwest (Ogun)	primary	Hospital: Different clinical samples	338	161	VAN, CPR, TET, ERY, OFL, GEN, COT, AUG, CAZ, CEF, CTR
[57]	Ibe et al., 2014	Southeast (Abia)	Primary	Hospital: Different clinical samples	84	69	MET
[58]	Onaolapo et al., 2016	Northwest(Kaduna)	Primary	Hospital: Wound and skin	65	22	VAN, CPR, ERY, AMP, AMO, CLIN, CEF, CTR
[59]	Ugwu et al., 2016	Southsouth (Delta)	Primary	Urban: Nasal	300	218	MET
[60]	Tula et al., 2016	Northeast	Primary	Hospital: Different clinical samples	100	45	CPR, AMO, OFL, GEN, CXC, CAZ, CRX, CTR
[61]	Anyanwu et al., 2013	Northwest (Kaduna)	Primary	Hospital: Skin	400	69	VAN, CHL, CAZ, CTR
[62]	Onyeagwara et al., 2014	Southsouth (Edo)	Primary	Hospital: Nasal	50	25	CPR, ERY, AMP, AMO, GENSTR, CAZ
[63]	Ngwai and Bakare, 2012	Northcentral (Nasarawa)	Primary	Urban: Urine	300	60	CHL, TET, ERY, AMO, GENCXC, STR
[64]	Umar et al., 2015	Nortwest (Kaduna)	Primary	Hospital: Skin and nasal	40	34	CPR, CHL, ERY, AMO, GEN, RIF, STR
[65]	Obajuluwa et al., 2015	Northwest (Kaduna)	Primary	Hospital: Wound and skin	100	39	VAN, CPR, ERY, AMP, AMO, GENCEF, CTR
[66]	Iduh et al., 2015	Southsouth	Primary	Hospital: Wound	300	64	TET, AMP, GEN, STR
[67]	Ibanga et al., 2020	Southsouth (Akwa-Ibom)	Primary	Hospital Different clinical samples	100	28	TET, CHL, ERY, AMO, GEN, STR
[68]	Emeakaroha et al., 2017	Southeast (Imo)	Primary	Urban: Nasal and throat	54	28	CHL, ERY, AMO, AMP, COT, CRX, PEN
[69]	Bisi-Johnson et al., 2005	Southwest (Oyo)	Primary	Hospital: Different clinical samples	86	97	TET, CHL, AMP, AMO, GENCXC, STR, PEN
[70]	Ayepola et al., 2015	Southwest (Lagos)	Secondary	Hospital:Nasal	`	217	TET, GEN, PEN
[71]	Odogwu et al., 2019	Northcentral (Abuja)	Primary	Hospital: Different clinical samples	360	55	CPR, ERY, AMP, GEN, RIF, CLIN, STR, TRIM
[72]	Adeiza et al., 2020	Northwest (Sokoto)	Primary	Hospital: Nasal	378	33	TET, CHL, ERY, GEN, CLIN, CAZ, CEF, TRIM
[73]	Ismail et al., 2015	Northeast (Borno)	Primary	Urban: Different clinical samples	110	42	CPR, CHL, ERY, AMO, GEN, RIF, STR, NOR
[74]	Ibrahim et al., 2018	Northwest (Kano)	Primary	Hospital: Wound and ear	150	71	CPR, TET, ERY, GEN, CLIN, CEF, TRIM, CTR
[75]	Olowe et al., 2013	Southwest (Ekiti)	Primary	Hospital: Different clinical samples		208	VAN, MET, TET, ERY, GEN, PEN, CEF
[76]	Oche et al., 2021	Northwest (Kano)	Primary	Hospital: Different clinical samples	140	26	MET, CPR, TET, ERY, AM, GEN, CEF, TRIM, NOR
[77]	Onelum et al., 2015	Southwest (oyo)	Primary	Hospital: Different clinical samples	246	102	MET, CHL, GEN, CLIN, CAZ, CEF
[78]	Akinduti et al., 2021	Southwest (Ogun)	Primary	Hospital: Different clinical samples	256	68	VAN, CPR, TET, ERY, AMO, OFL, GEN, CAZ, CRX, TRIM
[79]	Oladipo et al., 2019	Southwest (Osun)	Primary	Hospital: Different clinical samples		25	MET, CPR, ERY, AMO, GEN, OFL, CXC, CEF, CRX
[80]	Ogefere et al., 2020	Southsouth (Edo)	Secondary	Urban: Different clinical samples		556	MET
[81]	Motayo et al., 2012	Southwest (Ogun)		Hospital: Different clinical samples		50	MET, TET, CHL, ERY, AMO, GEN, CTR
[82]	Onyeka et al., 2021	Southsouth (Rivers)	Primary	Urban:	150	78	ERY, OFL, GENCXC, AUG, CAZ, CRX, CTR
[83]	Ugwu et al., 2009	Southeast (Enugu)	Primary	Nasal	100	53	TET, CHL, AMO, GEN, COT, AUG
[84]	Nsofor et al., 2016	Southeast (Abia)	Primary	Hospital: Different clinical samples	424	104	CPR, TET, CHL, ERY, AMP, CAZ, PEN
[85]	Mbim et al., 2017	Southsouth (Cross river)	Primary	Hospital: Nasal	150	42	MET, CPR, CHL, ERY, AMO, GEN, RIF, CEF, NOR
[86]	Ogbolu et al., 2015	Southwest (Osun)	Secondary	Hospital: Different clinical samples		116	VAN, TET, ERY, GEN, CAZ
[87]	Osinupebi et al., 2018	Southwest (Ogun)	Primary	Hospital: Different clinical samples	338	161	VAN, CPR, TET, ERY, OFL, GEN, COT, AUG, CAZ, CEF, CTR
[88]	Ajoke et al., 2012	Northcntral (Plateau)	Primary	Urban: Nasal	200	98	TET, ERY, AMP, AMO, GEN
[89]	Onyebueke et al., 2019	Southeast (Enugu)	Primary	Hospital: Urine	818	89	CPR, ERY, AMO, GEN, STR, NOR
[90]	Adetutu et al., 2017	Southwest (Ota)	Primary	Urban: Pimple	20	20	TET, CHL, ERY, GEN, CXC, COT, AUG, STR
[91]	Bale et al., 2021	Southwest (Kwara)	Primary	Hospital: Urine	856	56	MET, CPR, TET, CHL, ERY, AMO, OFL, GEN, AUG, CEF, CTR
[92]	Nmema, 2017	Southwest (Ondo)	Primary	Urban: Skin and nasal	80	34	ERY, GEN, CXC, AUG, CAZ, CRX, CTR
[93]	Ike et al., 2016	Southeast (Anambra)	Primary	Hospital: Nasal and hand	261	142	MET
[94]	Ugwu et al., 2015	Southeast (Anambra)	Primary	Hospital: Nasal	100	68	CPR, ERY, AMP, AMO, OFL, GEN, COT, STR, CTR
[95]	Emeka- Nwabunnia et al., 2015	Southeast (Imo)	Primary	Urban:Different clinical samples		59	VAN
[96]	Alli et al., 2012	Southwest (Osun)	Secondary	hospital: different samples		116	VAN, TET, ERY, AMO, GEN, CAZ
[97]	Sadauki et al., 2022	Northwest (Kano)	Primary	Hospital: Blood	214	40	MET, CPR, GEN, PEN, CTR
[98]	O’ Malley et al., 2015	Southwest (lagos)	Primary	Hospital: Different clinical samples	73	38	TET, ERY, GEN
[99]	Emeka- Nwabunnia et al., 2019	Southeast (Anambra)	Primary	Hospital: Different clinical samples	83	25	MET
[100]	Ako-Nai et al., 2005	Southwest (Osun)	Primary	Urban: Different clinical samples		112	CPR, TET, CHL, ERY, GEN
[101]	Frank-Peterside and Mukoro, 2010	Southsouth (Rivers)	Primary	Hospital: Different clinical samples		50	VAN, MET
[102]	Yahaya et al., 2022	Northwest (Kano)	Primary	Hospital: Different clinical samples	200	31	CPR, CHL, ERY, CLIN, COT, CEF
[103]	Onanuga et al., 2019	Southsouth (Bayelsa)	Primary	Urban: Nasal	390	47	CPR, TET, ERY, AMO, GEN, COT
[104]	Ogini and Olayinka, 2021	Southwest (Oyo)	Primary	Urban: Nasal	700	223	CPR, TET, ERY, AMO, GEN
[105]	Nwankwo et al., 2010	Northwest (Kano)	Secondary	Hospital: Different clinical samples		185	MET, CPR, AMO, OFL, GEN, CAZ, CTR
[106]	Olufunmiso et al., 2017	Southwest (Ogun)	Primary	Hospital: Different clinical samples	200	200	ERY, OFL, GEN, COT, AUG, CAZ, CRX, CTR
[107]	Olajide et al., 2012	Northwest (Kano)	Secondary	Hospital: Different clinical samples		100	ERY, AMO.CRX, NOR

*VAN* Vancomycin; *MET* Meticilin; *CPR* Ciprofloxacin; *TET* Tetracycline; *COT* Cotrimoxazole; *CHL* Chloramphenicol; *ERY* Erythromycin; *PEN* Penicillin; *CLIN* Clindmycin; *AMO* Amoxicillin; *AMP* Ampicillin; *GEN* Gentamycin; *CTR* Ceftriaxone; *AUG* Amoxicillin/clavulanic acid; *CAZ* Ceftazidime; *CRX* Cefuroxime; *CXC* Cloxacillin; *NOR* Norfloxacillin; *RIF* Rifampicin; *STR* Streptomycin; *OFL* Ofloxacin; *TRIM* Trimethroprim; *CEF* Cefoxitin

### Heterogeneity survey and publication bias

The included studies were conducted in the six geo-political zones of Nigeria; a total of 98 studies comprising of 26 from South South, 23 South West, 20 South East, 18 North West, 8 North Central and 3 North East. Quality assessment (risk of bias) was done in line with the following criteria: studies which used CLSI guideline for antibiotic resistant assessment, studies that used more than 20 *S. aureus* isolates and studies that used adequate sample representative of the region where testing was done. Agar diffusion based method was used to determine the resistance level of *S. aureus* isolates in all included studies. High heterogeneity was observed for each of the meta-analyses performed with I^2^ ranging from 79.36 to 98.60%; at p-values ≤ 0.01). This is due to vast difference in sample sizes; some studies used 20 isolates while some used 400 isolates which impacted on the resistance profile of each antibiotic. Also, number of clinical samples and recovered *S. aureus* isolates differ in all studies and these disparities resulted in high heterogeneity. More studies were conducted in the Southern (South South, South West, and South East) part of Nigeria giving rise to high heterogeneity. Studies were done in different hospitals within these regions with different prevalence estimates. Random sampling was used in most of the studies and different clinical samples were collected. More than one clinical sample per patient was collected in 51 studies while one clinical sample was collected per patient in 47 studies. Egger’s test for a regression intercept gave a p-value range of 0.06 to 0.99, indicating no evidence of publication bias (Additional file 2: S2) following Eggers’ test rule which state that ‘P-value less than 0.05 indicates the presence of publication bias’.

### Prevalence of *S. aureus* resistance to different antimicrobial agents

In this meta-analysis, the pooled prevalence of *S. aureus* resistance to twenty-three different antibiotics and the number of studies included in each meta-analysis is summarized in Table 2. Prevalence of resistance of *S. aureus* to each antibiotic based on pharmacological classification is given below for antibiotics routinely used in Nigeria.

**Table 2:** Pooled prevalence of *S. aureus* resistance to different antibiotics in Nigeria

Antibiotics	No. of studies	Total No. of isolates	No. of resistant isolates	Pooled AMR prevalence (95% CI)	I^2^(P-value) (P ≤ 0.01)
Vancomycin	29	2546	340	0.13 (0.7, 0.21)	96.60
Methicilin	30	3109	1445	0.46 (0.37, 0.56)	96.71
Ciprofloxacin	44	2739	838	0.31 (0.24, 0.38)	93.85
Tetracycline	43	3359	2170	0.65 (0.56, 0.76)	96.03
Cotrimoxazole	21	1293	855	0.66 (0.55, 0.76)	93.91
Chloramphenicol	32	2015	943	0.47 (0.37, 0.56)	95.03
Erythromycin	66	4969	2325	0.47 (0.40, 0.53)	95.31
Penicillin	15	1709	1396	0.82 (0.61, 0.96)	98.97
Clindamycin	12	787	275	0.35 (0.23, 0.49)	93.26
Amoxicillin	40	2167	1614	0.74 (0.66, 0.81)	94.64
Ampicillin	28	2074	1408	0.68 (0.53, 0.81)	97.91
Gentamycin	77	5470	1701	0.31 (0.25, 0.37)	95.90
Ceftriaxone	25	2144	943	0.44 (0.34, 0.54)	95.64
Amoxicillin/clavulanic acid	20	1665	1032	0.62 (0.50, 0.73)	95.76
Ceftazidim	24	2179	1329	0.61 (0.46, 0.75)	98.01
Cefuroxime	17	1035	714	0.69 (0.51, 0.85)	97.23
Cloxacillin	22	1565	1205	0.77 (0.64, 0.88)	97.13
Norfloxacillin	9	491	162	0.33 (0.17, 0.52)	95.27
Rifampicin	7	302	72	0.24 (0.06, 0.48)	95.19
Streptomycin	20	1287	579	0.45 (0.34, 0.57)	94.08
Ofloxacin	25	2058	494	0.24 (0.18, 0.31)	91.63
Trimethoprim	6	291	160	0.55 (0.35, 0.74)	91.99
Cefoxitine	21	1791	770	0.43 (0.31, 0.56)	96.61

### Prevalence of resistance *S. aureus* to rifamycins (rifampicins)

**Fig. 2:**
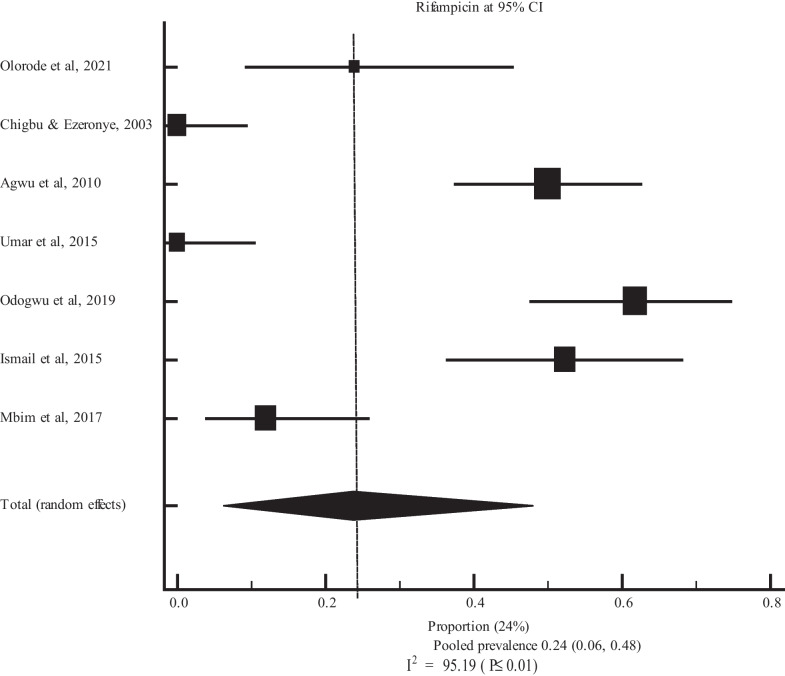
Forest plot of the prevalence of *S. aureus* resistance to rifampicin

Seven studies involving the prevalence of resistance to rifampicin was analyzed. The pooled prevalence of resistance of *S. aureus* to rifampicin in Nigeria is 24% (95% confidence interval [CI] 6%, 48%). The forest plot (rifampicin) is presented in Fig. 2.

### Prevalence of resistance of *S. aureus* to glycopeptides (vancomycin)

**Fig. 3:**
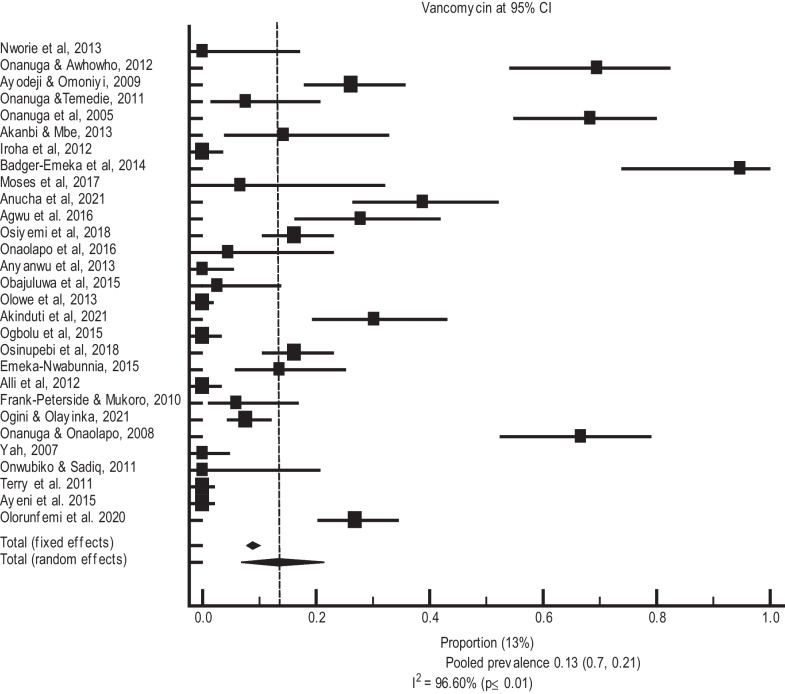
Forest plot of the prevalence of *S. aureus* resistance to vancomycin

The pooled prevalence of *S. aureus* resistance to vancomycin is 13% (95% CI 7%, 21%) and the forest plot is presented in Fig. 3. Sensitivity results after exclusion of four studies [20, 22, 27, 36] that reported high prevalence of *S. aureus* resistant to vancomycin is 7% (95% CI 3.3%, 12%). Hence, there was significant decrease in poled prevalence.

### Prevalence of resistance of *S. aureus* to beta-lactams antibiotics

**Fig. 4:**
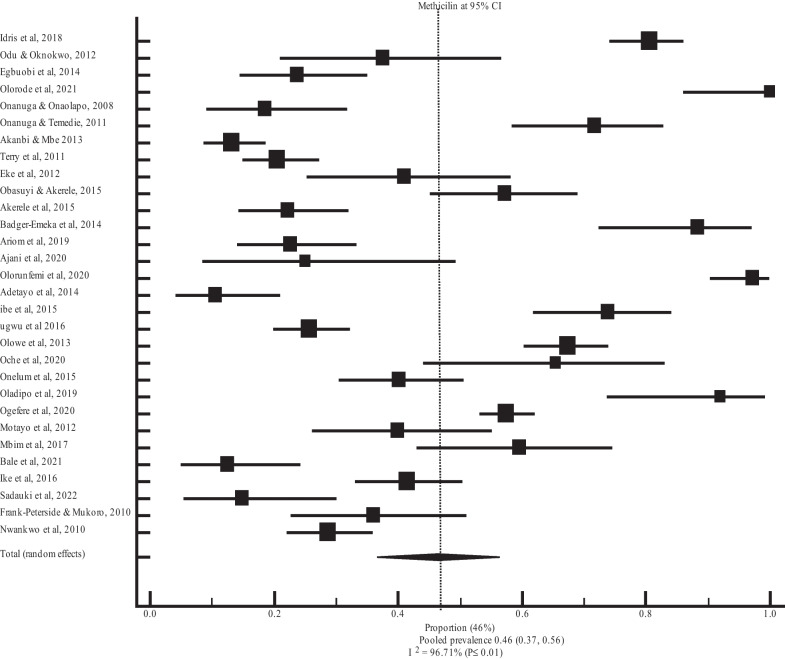
Forest plot of the prevalence of *S. aureus* resistance to methicillin

**Fig. 5:**
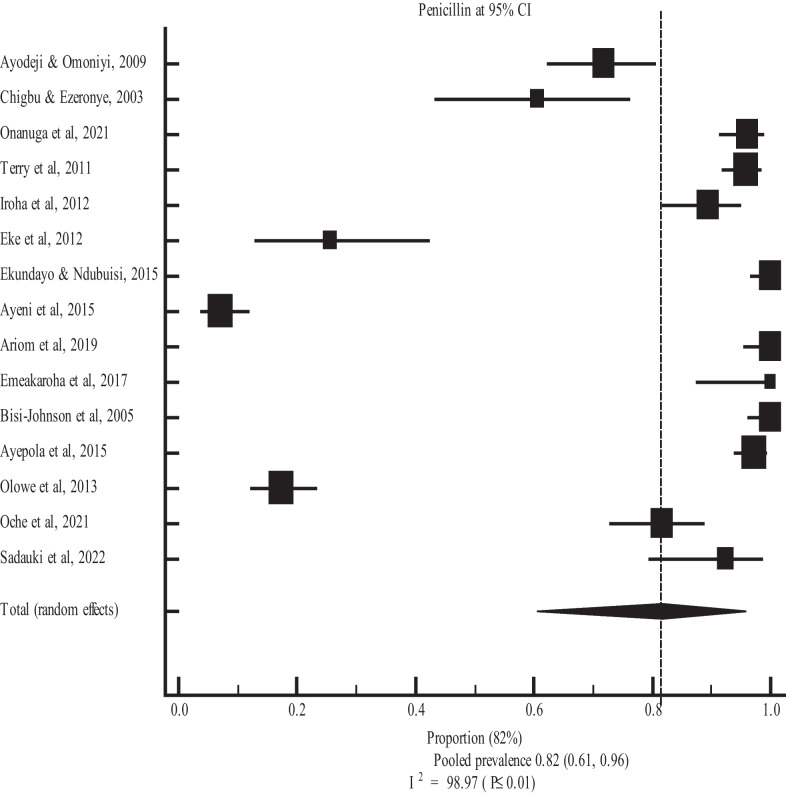
Forest plot of the prevalence of *S. aureus* resistance to penicillin G

Estimation of the pooled prevalence of *S. aureus* resistance to penicillin antibiotics (penicillin G, methicillin, amoxicillin, cloxacillin, ampicillin, and amoxacilin/caluvanic acid are here presented. Resistance to penicillin G, amoxicillin, cloxacillin, ampicillin, and augmentin were estimated based on 15, 40, 22, 28 and 20 studies respectively. Pooled prevalence resistance rates were highest in penicillin G at 82% (95% CI 61%, 96%). Resistance to cloxacillin [77% (95% CI 64%, 88%)], to amoxicillin [74% (95% CI 66%, 81%)], to ampicillin [68% (95% CI 53%, 81%)] and to amoxacilin/caluvanic [62% (95% CI 50%, 73%)]. However, resistance rate was moderate for methicillin [46% (95% CI 37%, 56%)]. Forest plots for antibiotics (methicillin and penicillin G) resistance are shown in Fig. 4 and 5, respectively while the forest plots for amoxicillin, ampicillin, amoxicillin/clavulanic acid and cloxacillin resistance are presented in Additional file 3: S3, Additional file 4: S4, Additional file 5: S5 and Additional file 6: S6 respectively.

**Fig. 6:**
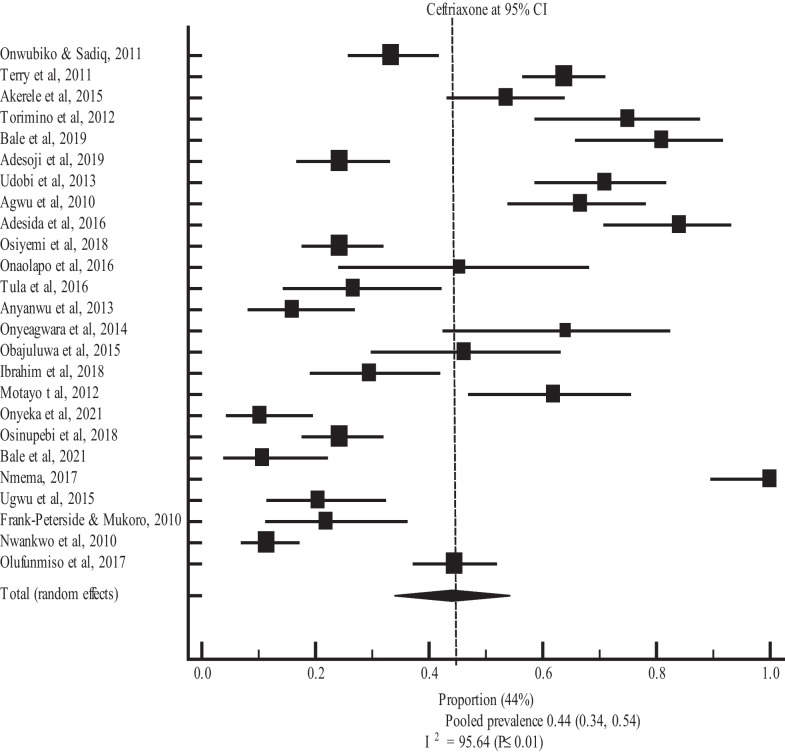
Forest plot of the prevalence of *S. aureus* resistance to ceftriaxone

Higher prevalence of resistance among cephalosporin antibiotic was observed in cefuroxime 69% (95% CI 51%, 85%) followed by ceftazidime 61% (95% CI 46%, 75%). Resistance to ceftriaxone is 44% (95% CI 34%, 54%) and to cefoxitine is 43% (95% CI 31%, 546%). The forest plot for ceftriaxone resistance is presented in Fig. 6 while the forest plots for cefuroxime and cefoxitine resistance are presented respectively in Additional file 7: S7 and Additional file 8: S8.

### Prevalence of resistance of S. aureus to floroquinolones

**Fig. 7:**
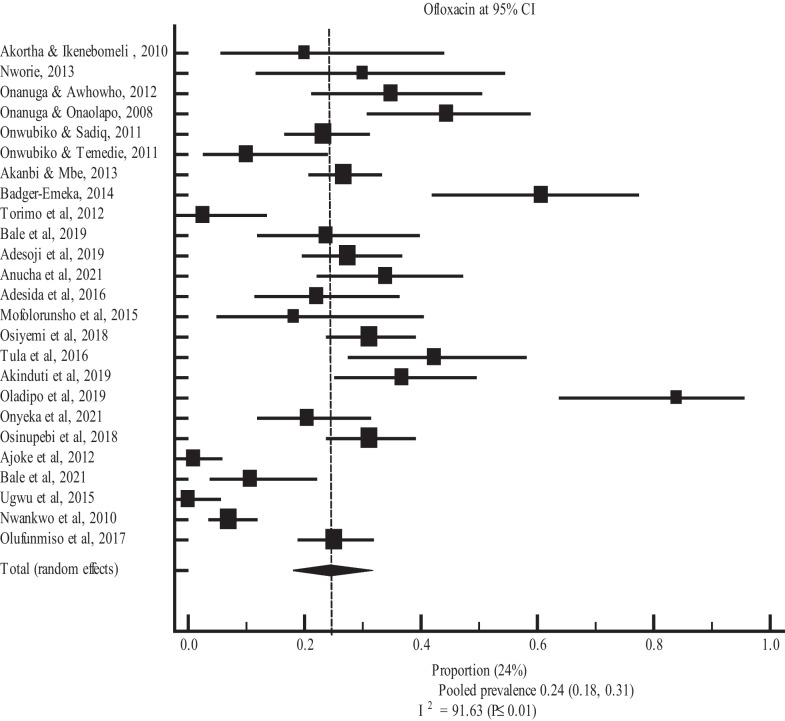
Forest plot of the prevalence of *S. aureus* resistance to ofloxacin

Three antibiotics (ciprofloxacin, ofloxacin, and norfloxacilin) from floroquinolones were included in the study. For ciprofloxacin, 44 studies were used to estimate the pooled resistance, 25 were used for ofloxacin and 9 studies were used for norfloxacilin. The pooled prevalence of resistance of *S. aureus* to ciprofloxacin [31% (95% CI 24%, 38%)], ofloxacin [24% (95% CI 18%, 31%)], and to norfloxacillin [33% (95% CI 17%, 52%)]. The forest plot for ofloxacin resistance is presented in Fig. 7 while the forest plot for ciprofloxacin and norfloxacilin included in Additional file 9: S9 and Additional file 10: S10.

### Prevalence of resistance of *S. aureus* to protein synthesis inhibitors

**Fig. 8:**
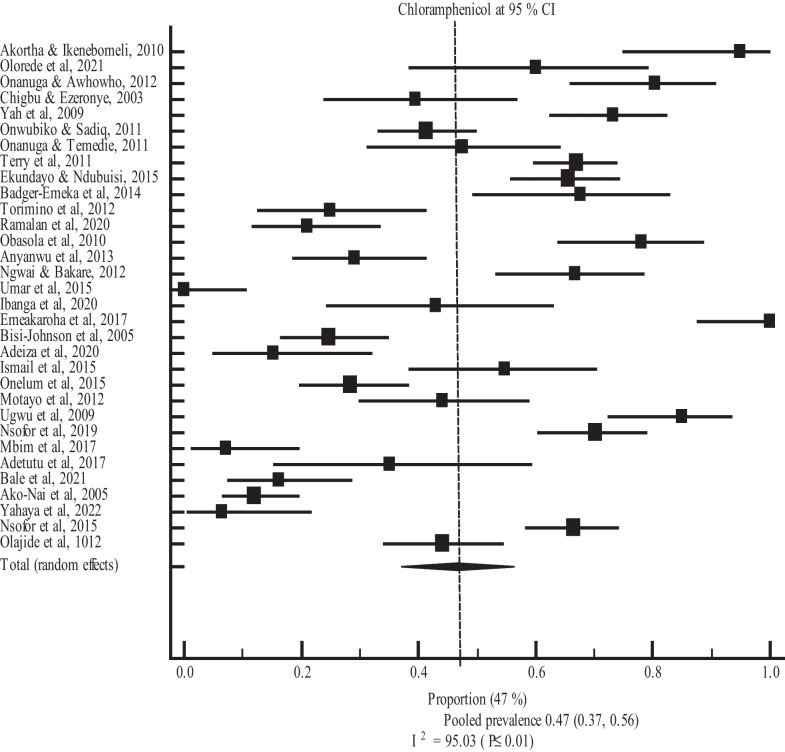
Forest plot of the prevalence of *S. aureus* resistance to chloramphenicol

Tetracycline a reversible protein synthesis inhibitor showed the highest resistance rate [65% 995% CI 56%, 76%)] followed by erythromycin (macrolides) [47% (95% CI 40%, 53%)] and chloramphenicol [47% (95% CI 37%, 56%)], respectively. Aminoglycosides (gentamycin and streptomycin) and lincosamides (clindamycin) showed relatively lower level of resistance. The pooled prevalence of resistance to streptomycin [45% (95% CI 34%, 57%)], to clindamycin [35% (95% CI 23%, 49%)] and to gentamycin [31% (95% CI 25%, 37%)]. The forest plot for chloramphenicol resistance is presented in Fig. 8 while the forest plots for tetracycline, erythromycin, gentamycin, streptomycin, and clindamycin resistance are presented in Additional file 11: S11, Additional file 12: S12, Additional file 13: S13, Additional file 14: S14, and Additional file 15: S15 respectively.

### Prevalence of resistance of *S. aureus* to antimetabolites

**Fig. 9:**
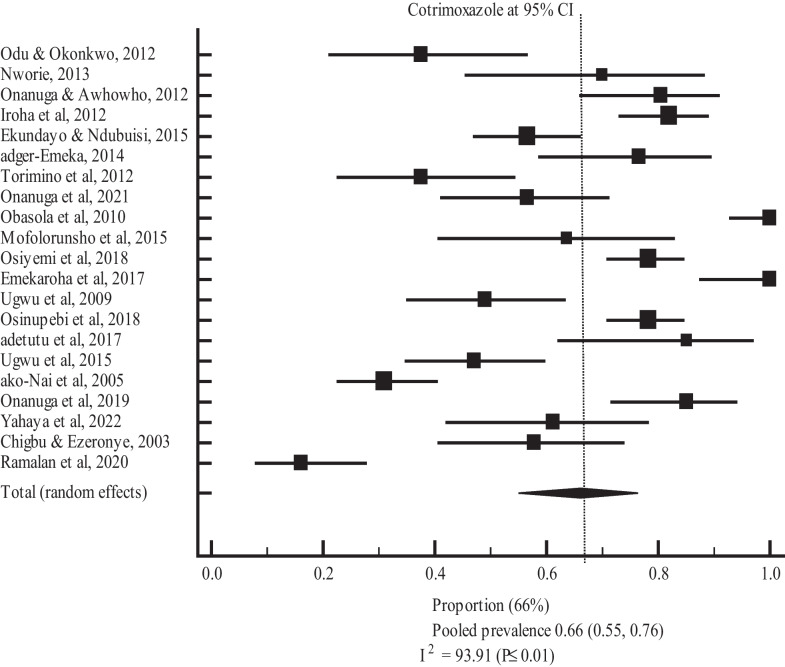
Forest plot of the prevalence of *S. aureus* resistance to cotrimoxazole

High resistance was observed among the antimetabolites antibiotics. Pooled prevalence of *S. aureus* resistance to cotrimoxazole was found to be 66% (95% CI 55%, 76%) and to trimethoprim is 55% (95% CI 35%, 74%). The forest plot for cotrimoxazole resistance is presented in Fig. 9 while the forest plot for trimethoprim is presented in Additional file 16: S16.

### Comparison of the prevalence of *S. aureus* resistance to different antibiotics

**Fig. 10:**
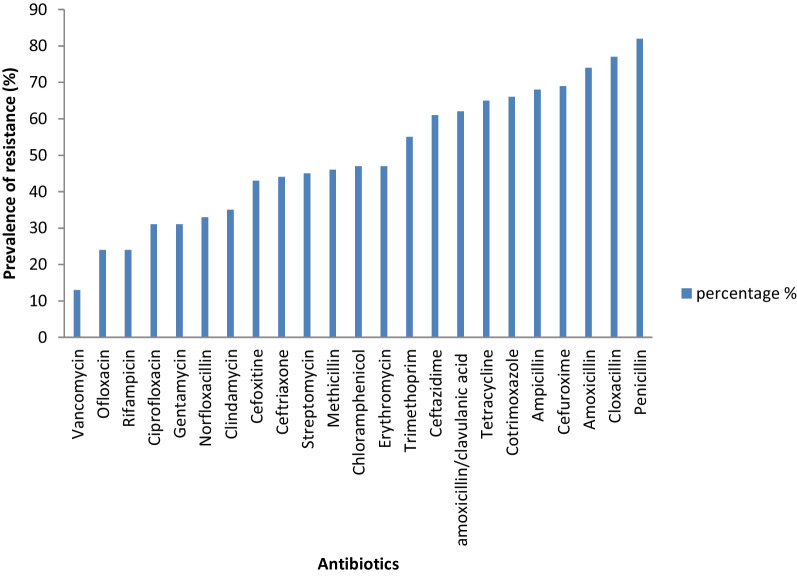
Comparison of the prevalence of *S. aureus* resistance to different antibiotics in Nigeria

The trend of prevalence of *S. aureus* resistance to different antibiotics addressed in this meta-analysis is shown in Fig. 10. From observation, the prevalence of resistance of *S. aureus* to the different antibiotics in this study ranges from 13 (vancomycin) to 82% (penicillin G).

The order of resistance in increasing order based on the pooled prevalence of *S.aureus* resistance to different antibiotics was observed to be vancomycin, ofloxacin, rifampicin, ciprofloxacilin, gentamycin, norfloxacillin, clindamycin, cefoxitine, ceftriaxone, streptomycin, methicillin, chloramphenicol, erythromycin, trimethoprim, ceftazidim, amoxicillin-clavulanic acid, tetracycline, cotrimoxazole, ampicillin, cefuroxime, amoxacilin, cloxacillin, and pencilin G.

## Discussion

Antimicrobial resistance continues to be on the rise which constitutes a serious public health problem globally. Many microbes have developed resistance to many different antimicrobial agents over time. This meta-analysis estimated the pooled prevalence of resistance of *Staphylococcus aureus* to 23 different antibiotics routinely used in Nigeria. Ninety eight studies [98] were included in this meta-analysis study with variation in the number of studies included in each meta-analysis which ranged from 6 to 77. In general, the 98 studies evaluated the rate of *S. aureus* resistance to different antibiotics based on 46,640 isolates of which 23, 048 were resistant to various antibiotics. Prevalence of resistance of *S. aureus* to different antibiotics ranges from 13 to 82%. Results from the meta-analysis showed that resistance of *S. aureus* to routinely used antibiotics in Nigeria was alarmingly high. From the studies, it was found that 82% *S. aureus* were resistant to penicillin G. However, it was observed from the studies that 24% of *S. aureus* were resistant to ofloxacin and rifampicin. In general, clinical samples (nasal, urine, wound, pimple, ear, blood, and vaginal swab) were collected from both symptomatic patients [61] and asymptomatic people [37].

High heterogeneity was observed for each of the meta-analyses performed with I^2^ ranging from 79.36 to 98.90% at p-values ≤ 0.01). This is because many studies used varying number of isolates/sample sizes. Some studies used 20 isolates while some used 400 isolates which impacted on the resistance profile of each antibiotic. This can better be illustrated in the prevalence of resistance of *S. aureus* to vancomycin. Sensitivity test was carried out to by removing studies that reported very high prevalence of *S. aureus* to vancomycin and the overall pooled prevalence reduced from 13 to 7%. This showed the degree of heterogeneity among studies. Possible cause of heterogeneity is due to different number of clinical samples and number of isolates recovered which were subjected to antibiotic sensitivity tests. Also random sampling of clinical samples can also be the possible cause. Publication bias was evaluated for all meta-analysis of the 23 antibiotics and publication bias was not found. Egger test is use to estimate asymmetry of data using funnel plots. p-value less than 0.05 using Egger criteria indicate no presence of publication bias even though erythromycin had p-value of 0.017 which is below 0.05. This is because a p-value of 0.017 for the Egger test means that the results found have a 1.7% chance to occur when there is no 'small sample bias.

The pooled prevalence of *S. aureus* resistance to Beta-lactams class of antibiotics was extremely high especially for penicillins. *S. aureus* showed highest resistance to penicillin G (82%) and 69% resistance to cefuroxime (cephalosporin). The pooled estimate of *S. aureus* resistance to penicillin G is comparable with the reported estimation of worldwide resistance of 90–95% [108]. This is not surprising due to the fact that penicillin G is the first antibiotic to be discovered. Bacteria are able to develop resistance to antibiotics due to selective pressure from antibiotics. Selective pressure from penicillin led to the production of beta-lactamase to conuter the effect of beta-lactam antibiotics. Consequently, semi-synthetic beta-lactam antibiotics such as ampicillin, Amoxicillin/clavulanic acid and amoxicillin with different side chains were developed to counter such bacteria strains. However, *S. aureus* resistance to amoxicillin and ampicillin is relatively high from our results. Lower rate of resistance was observed among beta-lactamase-resistant antibiotics (methicillin, ceftriaxone, cefoxitine). Also, lower rate of resistance to clindamycin might be attributed to infrequent use of the antibiotic. Amoxicillin-clavulanic acid was developed as a combination of an antibiotic (amoxicillin) and non-antibiotic (clavulanic acid). Clavulanic acid inhibit beta-lactamase enzyme which prolong the antibacterial activity of amoxicillin component; however, results from the meta-analysis showed high resistance of *S. aureus* to amoxicillin/clavulanic acid.

Another semi-synthetic penicillin resistant antibiotic called methicillin was developed which is resistant to hydrolysis of beta-lactamase was developed. The term Methicillin Resistant Staphylococcus aurues (MRSA) is synonymous with multi-drug resistance (MDR) because MRSA are invariably resistant to different antibiotics. Acquisition of *mec* A gene that encodes penicillin binding protein confers resistance to *S. aureu* [109]. The pooled prevalence of *S. aureus* to methicillin (46% [95% CI 37%, 56%]) in Nigeria is similar to 2014 global surveillance reports of the world health organization (WHO) [110] 2014. Which depicted MRSA prevalence ranged 33–95% in Africa. Similarly, the pooled estimate of 46% in our study is also in agreement with the pooled prevalence estimate of MRSA in continents such as North America, Asia, and Europe which ranges from 23.1 to 47.4% [109]. The high pooled prevalence in our study might be due to certain factors and variables such as the inclusion of nosocomial and community acquired infections in the original studies analyzed. Generally, nosocomial infection causing pathogens are believed to possess higher resistance rate due to prolonged and higher exposure to different antimicrobial agents and exchange of genetic materials. Thus, there is greater transmission of resistant genes through various means within the hospital settings [111]. The implication of infections cause by MRSA is difficulty in treatment which often requires alternative antimicrobial agents which are most times very expensive.

The pooled prevalence of *S. aureus* resistance to vancomycin (13% at 95% CI [0.7%, 21%]) in this meta-analysis is high and a cause for concern when compared to global prevalence estimate [4]. The prevalence of vancomycin resistant *S. aureus* (VRSA) in Africa was reported to be 2.5% [4]. This is quite low when compared to the result from this study which is very high (13%). With this increased resistance, the use of vancomycin to treat MRSA is becoming problematic and poses serious health challenge. The rise in VRSA might be due to the indiscriminate use of vancomycin in Nigeria. By the way, Four studies [20, 22, 28, 36] reported a very high prevalence of VRSA; however, sensitivity analysis showed that they had high significant influence on the overall pooled prevalence estimate. Removing the three studies reduces the pooled prevalence of *S. aureus* resistance to vancomycin from 13 to 7%. Analyzing studies that depicted high prevalence of resistance of *S. aureus* to vancomycin showed that the same author conducted and published the three studies in peer reviewed journals. Urine samples were mainly used for *S. aureus* isolation by the author in the three studies of which [20, 22] were from symptomatic urinary tract infection patients who visited the hospitals and [27] from healthy volunteers. Urinary tract infection is a common infection and a reason for antibioticl use; consequently, resistant microbial strains have emerged. This reason might be attributed to the high prevalence of *S. aureus* resistant to vancomycin in the three studies. Exposure to resistant strains especially in hospital settings might have resulted in the increased resistance to vancomycin in the three studies [112, 113]. This is because in Nigeria, expired or waste antibiotics are not properly discharged. This could result in selective pressure on inhabitant microorganisms which results in development of various resistant mechanisms.

Generally, the global pattern of antimicrobial resistance varies among different geographical locations and socioeconomic level [114, 115]. Variations in studies can be attributed to design, time, and population involved. Heterogeneity tests at p ≤ 0.01 showed significant variation among included studies in this meta-analysis. Therefore, it is reasonable to assert that the study population might be infected with the same strains of *S. aureus* within the same location at a specified period. This is because most of the studies were conducted within a specified period of time and area.

Mechanisms of resistance of *S. aureus* include: production of beta-lactamase enzymes to deactivate beta-lactam sensitive antibiotics, efflux pump for extruding antibiotics such as tetracyclines [6], reduced accumulation of macrolides antibiotics [7], production of aminoglycoside modifying enzymes to inactivate aminoglycosides antibiotics, alteration of DNA gyrase and topoisomerase IV expression for floroquinolones antibiotics, and expression of Mec genes which alters penicillin binding proteins. From the results and mechanism of resistance of *S. aureus*, it can be said that *S. aureus* found in Nigeria are highly resistant to the beta-lactam class of antibiotics.

The pooled prevalence of *S. aureus* resistance to the floroquinolones class of antibiotics such as ciprofloxacin, ofloxacin, and norfloxacin was lower especially for ciprofloxacin which is commonly used within Nigeria. However, high pooled prevalence of *S. aureus* resistance to antimetabolites class of antibiotics (cotrimoxazole and trimethoprim) was observed.

From the meta-analysis, *S. aureus* mediated infection in Nigeria can be treated using vancomycin, floroquinolones, and aminoglycosides. MRSA has been a concern in Nigeria especially with the incidence of VRSA. Newer alternative antibiotics such as linezolid, telavancin, ceftaroline, tigecycline and daptomycin are rarely used in Nigeria. Various factors such as lack of infection prevention which lead to reoccurrence of infection, inappropriate use of antibiotics, poor hospital facilities, lack of routine susceptibility test before antibiotic administration, and self medication contributes to the rapid emergence and re-emergence of AMR. Tackling this factors, will go a long way in the fight against the continue rise of MDR pathogens in general.

### Study limitations

Most of the included studies share similar characteristics. The search was limited to only titles that deal with antibiotic resistance. Selection was done randomly especially in Google Scholar with had 35, 400 studies results from the search. The meta-analysis was done once for each antibiotics and sub-grouping to reduce high heterogeneity and publication bias was not done due to too many meta-analysis already done. The included studies used in-vitro antimicrobial assays which has limitations such as difficulties in interpreting data, variability of testing media (differences in cation content, acidic or alkaline), and difficulty in knowing the pharmacokinetics of an antibiotic or post effect of an antibiotic (a situation where bacteria growth is inhibited even when the antibiotic concentration falls below the MIC). Most of the studies were done in teaching hospitals and tertiary institutions in big cities; hence both symptomatic and asymptomatic individuals are involved. For symptomatic individuals, most of the studies were done in teaching hospitals were patients with chronic and recurrent infections are treated; resistance level could be overestimated.

## Conclusion

The results of this meta-analysis showed that *S. aureus* is resistant to many routinely used antibiotics in Nigeria. It is highly resistant to beta-lactams, tetracyclines, and antimetabolites antibiotics. Resistance of *S. aureus* to vancomycin remains a serious health problem due to limited treatment options. There is a lot of variation in resistance estimates between studies. High heterogeneity was observed in each meta-analysis for each antibiotic which was attributed to various factors such as different clinical sample and recovered isolates sizes, random sampling and method used for resistance investigation. Hence it is imperative to develop programs to promote rational use of antimicrobial agents, infection prevention and control to reduce the incidence of AMR. In addition, furthers researches focusing on identifying the dynamics promoting microbial resistance, infectious microbial strains and molecular/genetic basis of resistance should be encouraged.

## Supplementary Information

Additional file 1: S1. PRISMA 2009 Checklist

Additional file 2: S2. Egger’s test of publication bias

Additional file 3: S3. Forest plot of the prevalence of S. aureus resistance to amoxicllin

Additional file 4: S4. Forest plot of the prevalence of S. aureus resistance to ampicillin

Additional file 5: S5. Forest plot of the prevalence of S. aureus resistance to augmentin

Additional file 6: S6. Forest plot of the prevalence of S. aureus resistance to cloxacillin

Additional file 7: S7. Forest plot of the prevalence of S. aureus resistance to cefuroxime

Additional file 8: S8. Forest plot of the prevalence of S. aureus resistance to cefoxitine

Additional file 9: S9. Forest plot of the prevalence of S. aureus resistance to ciprofloxacin

Additional file 10: S10. Forest plot of the prevalence of S. aureus resistance to norfloxacin

Additional file 11: S11. Forest plot of the prevalence of S. aureus resistance to tetracycline

Additional file 12: S12. Forest plot of the prevalence of S. aureus resistance to erythromycin

Additional file 13: S13. Forest plot of the prevalence of S. aureus resistance to gentamycin

Additional file 14: S14. Forest plot of the prevalence of S. aureus resistance to streptomycin

Additional file 15: S15. Forest plot of the prevalence of S. aureus resistance to clindamycin

Additional file 16: S16. Forest plot of the prevalence of S. aureus resistance to trimethoprim

## Data Availability

The data supporting the conclusions of this article are included within the article and its supporting information.

## Abbreviations

AMR: Antimicrobial resistance
CLSI: Clinical Laboratory Standard Institute
CI: Confidence interval
MRSA: Methicillin resistant *Staphylococcus aureus*
*S. aureus*: *Staphylococcus aureus*
VRSA: Vancomycin resistant *Staphylococcus aureus*
