# Supplementary material for: A meta-analysis on the prevalence of resistance of Staphylococcus aureus to different antibiotics in Nigeria
Source: Antimicrob Resist Infect Control. 2023 Apr 25;12:40. doi: 10.1186/s13756-023-01243-x (PMC10127087; doi:10.1186/s13756-023-01243-x)
Supplement: Supplementary file 2 — Additional file 2: S2. Egger’s test of publication bias [file 13756_2023_1243_MOESM2_ESM.docx]

**Additional file 2 S2.** Egger’s test of publication bias

| No. | Antimicrobial | Egger’ test | P |
| --- | --- | --- | --- |
| 1 | Vancomycin | -0.23 | 0.06 |
| 2 | Methicilin | 0.57 | 0.826 |
| 3 | Ciprofloxacin | 0.61 | 0.769 |
| 4 | Tetracycline | 1.74 | 0.490 |
| 5 | Cotrimoxazole | 0.17 | 0.954 |
| 6 | Chloramphenicol | -1.36 | 0.622 |
| 7 | Erythromycin | 4.07 | 0.017 |
| 8 | Penicillin | 5.81 | 0.537 |
| 9 | Clindamycin | 5.42 | 0.143 |
| 10 | Amoxicillin | 1.62 | 0.514 |
| 11 | Ampicillin | 1.27 | 0.753 |
| 12 | Gentamycin | -1.90 | 0.302 |
| 13 | Ceftriaxone | 5.31 | 0.094 |
| 14 | Augumentin | 1.37 | 0.732 |
| 15 | Ceftazidim | 6.16 | 0.196 |
| 16 | Cefuroxime | -1.53 | 0.781 |
| 17 | Cloxacillin | 0.01 | 0.998 |
| 18 | Norfloxacillin | 7.60 | 0.090 |
| 19 | Rifampicin | -18.38 | 0.143 |
| 20 | Streptomycin | -0.180 | 0.952 |
| 21 | Ofloxacin | 1.53 | 0.464 |
| 22 | Trimethoprim | -3.44 | 0.720 |
| 23 | Cefoxitine | 2.79 | 0.430 |
