# Supplementary material for: A meta-analysis on the prevalence of resistance of Staphylococcus aureus to different antibiotics in Nigeria
Source: Antimicrob Resist Infect Control. 2023 Apr 25;12:40. doi: 10.1186/s13756-023-01243-x (PMC10127087; doi:10.1186/s13756-023-01243-x)
Supplement: Supplementary file 3 — Additional file 3: S3. Forest plot of the prevalence of S. aureus resistance to amoxicllin [file 13756_2023_1243_MOESM3_ESM.docx]

**Additional file 3 S3**. Forest plot of the prevalence of *S. aureus* resistance to amoxicllin
